# Chronic Disease Self-Management Support: Public Health Perspectives

**DOI:** 10.3389/fpubh.2014.00234

**Published:** 2015-04-27

**Authors:** Teresa J. Brady, Lynda A. Anderson, Rosemarie Kobau

**Affiliations:** ^1^Arthritis Program, National Center for Chronic Disease Prevention and Health Promotion, Centers for Disease Control and Prevention, Atlanta, GA, USA; ^2^Healthy Aging Program, National Center for Chronic Disease Prevention and Health Promotion, Centers for Disease Control and Prevention, Atlanta, GA, USA; ^3^Rollins School of Public Health, Emory University, Atlanta, GA, USA; ^4^Epilepsy Program, National Center for Chronic Disease Prevention and Health Promotion, Centers for Disease Control and Prevention, Atlanta, GA, USA

**Keywords:** self-management support, evidence-based interventions public health, life course, prevention research

## A Public Health Priority

The Centers for Disease Control and Prevention (CDC) has a longstanding commitment to developing and promoting evidence-based strategies to prevent or delay disease and disability (1, 2). Significant among these strategies is support for self-management of chronic diseases. About one-half of all U.S. adults have at least one chronic condition (3) and over two-thirds of Medicare beneficiaries aged 65 years or older have two or more chronic conditions (4). Given that the risk of developing a chronic disease increases with advancing age (5), the dramatic aging of the U.S. population underscores the importance of chronic disease self-management supports. Further, effective self management of chronic conditions is essential to achieving a state of health, which is proposed to reflect “the ability to adapt and to self manage” (6).

An effective approach to improve population health requires a strong focus on self-management. CDC’s National Center for Chronic Disease Prevention and Health Promotion includes among its four priorities efforts to help ensure that “communities support and clinics refer patients to programs that improve management of chronic conditions” (7). Self-management (e.g., what individuals and families do on a daily basis to feel better and pursue the life they desire) (8) and self-management support (e.g., actions taken by others to support individual self-management) (9) are critical strategies in meeting this priority objective. The U.S. Department of Health and Human Services recognized the importance of self-management support in its framework for addressing multiple chronic conditions (MCC). One of the four goals of the framework is to “maximize the use of proven self-care management and other services by individuals with MCC” (10).

Chronic disease self-management support occurs at the intersection of public health, clinical healthcare delivery, social services, aging services networks, and other community resources. In this commentary, we provide a public health perspective on self-management support, identify examples of CDC investment in self-management support activities, and discuss potential future directions. These examples are provided to illustrate the breadth of CDC’s work in this area and are not designed to serve as comprehensive list of CDC’s investment in self-management support.

## An International Foundation for Understanding

Consistent with a public health perspective, we advance an expanded definition of self-management support from the International Framework for Chronic Condition Self-Management Support. This definition describes self-management support as a grouping of policies, programs, services, and structures that extend across healthcare, social sectors, and communities to support and improve the way individuals manage their chronic conditions (11). The definition frames self-management support within a social-ecological perspective underscoring individual, interpersonal, community, environmental, and systems levels resources (12). This definition also embraces a life course perspective that attends to individual autonomy and decision-making as well as role changes and other adaptations to life events (13).

Self-management support takes many forms. It includes interventions such as the Chronic Disease Self-Management Program (CDSMP) (14) and the falls prevention programs featured in this special issue (15). It also includes supportive interactions between healthcare providers and patients, proactive follow-up, and social and physical environments that support healthy behaviors such as having safe places to exercise, access to healthy foods, and social norms that combat stigma, promote social participation, and support self-care behaviors (9).

Self-management support interventions are provided in a variety of formats (e.g., one-to-one, small groups, telephone, online/mobile, self-study); and in a variety of settings (e.g., home, healthcare, worksite, community) (9, 12). Although the form and formats vary, the goal of self-management support is consistent: to help individuals and their personal support system acquire and maintain the knowledge, skills, and confidence to do what they need to do to live as well as possible with their chronic condition(s).

## Advancing the Study and Application of Self-Management Support

The International Framework for Chronic Condition Self-Management Support identifies seven key strategic directions to move self-management support forward in research, policy, and practice at the local, regional, state, and national levels. These strategic directions are to involve consumers, expand the reach and range of services, advance evidence, improve effectiveness and appropriateness of services, strengthen inter-sector linkages, foster multi-sector commitment and accountability, and build infrastructure. Using this organizing structure (11), in Table 1, we highlight a few select but illustrative examples of CDC’s contributions to the Framework’s seven strategic directions.

**Table 1:** **Self-management support strategic directions: select CDC program examples**.

Strategic direction	Tactic	CDC program examples^a^
Involve consumers	Community-based participatory research	CDC’s prevention research centers (PRC) use community-based participatory research methods as a foundation for their research (16). Evidence-based intervention programs such as Enhance Fitness (17) to increase physical activity among older adults and PEARLs (18) to screen and treat depression among older adults were developed at PRCs (http://www.cdc.gov/prc).
	Audience research	CDC collaborated with the Arthritis Foundation to support qualitative and quantitative market research that provided insights into the types of services people with arthritis want to support their self-management efforts (Listening to Consumers: What do People With Arthritis Want? A Focus Group Report to: The Centers for Disease Control and Prevention (Arthritis Program) and the Arthritis Foundation. Unpublished report by Fleishman Hillard, 2006; Receptivity to Existing and Potential Programs and Services for People with Arthritis. A Report to the Arthritis Foundation. Unpublished report by Fleishman Hillard, 2007).
		CDC conducted audience research with people with various chronic conditions and determined that non-disease specific, non-intervention specific messaging to increase the visibility of self-management education would resonate with consumers and motivate them to seek more information on specific interventions (Audience Research to Determine the Feasibility of Developing a Marketing Campaign to Increase Visibility of Self-Management Education. Unpublished report submitted to CDC by FHI 360, 2014). CDC is developing this type of broad awareness campaign.
Expand reach and range of services	Tele-health	Emory University developed Project UPLIFT, an effective tele-health intervention delivered by phone and Internet that helps adults with epilepsy and comorbid depression reduce their depressive symptoms, and improve some well-being domains (19).
	Self-study interventions	The University of North Carolina and Stanford University developed and evaluated *The Arthritis Toolkit* that provides the content of the small group-delivered Arthritis Self-Management Program (ASMP) in a mail-delivered, self-study format (20). Currently, CDC is funding Stanford University to develop a self-study version of the Chronic Disease Self-Management Program (CDSMP).
	Online interventions	Stanford University^b^ developed an online (virtual group) version of the ASMP (21), and national dissemination^a^ is being pilot-tested by the Arthritis Foundation.^a^ (http://www.arthritistoday.org/arthritis-self-management-program/).
		The American Cancer Society pilot-tested a cancer-specific online version of the CDSMP, titled Cancer: Thriving and Surviving, developed at Stanford University (https://cancer.selfmanage.org/survivor/hl/hlMain).
		Emory University PRC developed and tested, WebEase (Epilepsy Awareness Support and Education), an online program that is available on the national Epilepsy Foundation web site (22).
Advance evidence	Meta-analysis	CDC conducted meta-analyses of 24 ASMP and 23 CDSMP studies that documented robust improvements in health outcomes and health behaviors across multiple studies (23, 24).
	Intervention research	PRCs, in collaboration with other universities, conducted effectiveness studies that substantiated evidence-based community interventions such as the Arthritis Foundation Exercise Program, Walk with Ease, Enhance Fitness, and First Steps to Active Health (http://www.cdc.gov/arthritis/funded_science/completed/index.htm).
Improve effectiveness and appropriateness of services	Clinical-decision support tools	The University of Texas (Houston) PRC developed and tested MINDSET (Management Information & Decision Support Epilepsy Tool), a tablet-based tool for inputting data on (1) seizures (e.g., history, management); (2) medicine (e.g., barriers, side effects), and (3) lifestyle (e.g., social support). The tool is designed to enhance patient-provider communication and action planning to sustain or improve epilepsy self-management behaviors (25).
	Comparative effectiveness studies	The University of North Carolina PRC conducted a study of CDSMP and ASMP among people with arthritis that documented the equivalence of these two interventions for people with arthritis (26).
		The University of Pittsburg PRC conducted a study of preventing falls among older adults that compared 3 strategies: usual care, an education program, or an education-plus-exercise program (http://www.caph.pitt.edu/wps/docs/falls/FP_AlbertfallsCDCpresentation9-25-11.pdf).
Strengthen inter-sector linkages	Community-clinical linkages	CDC’s Heart Disease and Stroke Prevention Program is collaborating with the Vermont Blue Print for Health to explore the use of community-health workers as part of the primary care team to help assess patients’ needs and coordinate community-based support services (27).
	Linking public health and aging services networks	The Healthy Aging Research Network, a thematic network of the PRC Program, developed and implemented a national research and dissemination agenda related to the public health aspects of healthy aging (28).
	Linking mental health and public health	The Managing Epilepsy Well Network, a thematic network of the PRC Program, includes interdisciplinary teams of researchers who collaborate across mental health and epilepsy sectors to develop and implement evidence-based self-management programs that target both physical and mental health needs of people with epilepsy (29).
	Linking multiple stakeholders	The Osteoarthritis Action Alliance, under the auspices of the Arthritis Foundation, provides a forum for multiple organizations to work collaboratively to advance the osteoarthritis public health agenda including increasing physical activity and fostering self-management education (http://www.oaaction.org).
Foster multi-sector commitment and accountability	Strategic frameworks	The U.S. Department of Health and Human Services developed the document, *The Multiple Chronic Conditions: A Strategic Framework* that outlines national strategies for improving health and quality of life for individuals with multiple chronic conditions (MCC), and cites the use of proven self-care management and other services by individuals with MCC as one of its’ four strategic goals (10).
		CDC staff participated in the development of the International Framework for Chronic Condition Self-Management Support that highlights priority strategic directions to advance the research, policy, and practice of self-management support (11).
	National objectives	Healthy People 2020 includes objectives to increase participation in self-management education among select chronic disease populations including people with arthritis and diabetes (http://www.healthypeople.gov/2020/topicsobjectives2020/objectiveslist.aspx?topicId=3; http://www.healthypeople.gov/2020/topicsobjectives2020/objectiveslist.aspx?topicId=8).
	Convening stakeholders	CDC collaborated with the Arthritis Foundation to convene a broad stakeholder group that developed *Environmental and Policy Strategies to Increase Physical Activity among People with Arthritis;* this document recommends strategies for action in 6 sectors including community, business, healthcare, transportation, parks and recreation, and mass media (http://www.arthritis.org/files/documents/OA_Physical_Activity_Rpt_508_v1_TAG508.pdf).
Build infrastructure	Professional training opportunities	The Managing Epilepsy Well Network provided professional training that helped providers better understand self-management strategies and how to implement at least three evidence-based self-management programs (http://web1.sph.emory.edu/ManagingEpilepsyWell/index.php).
	Capacity building	All 50 states receive CDC funding to support the delivery of diabetes self-management education through consolidated chronic disease funding (http://www.cdc.gov/chronicdisease/about/statepubhealthactions-prevcd.htm).
		Individual CDC programs focused on asthma, arthritis, diabetes, and heart disease have supported at least 40 state health departments to disseminate CDSMP.
		The National Association of County and City Health Officials, the National Recreation and Parks Association, and the Y-USA are testing their ability to serve as national delivery systems in disseminating self-management support interventions. http://naccho.org/topics/HPDP/chronicdisease/cdsmp.cfm; http://c.ymcdn.com/sites/www.chronicdisease.org/resource/resmgr/Arthritis_Monthly_Reports/030513_ACHandoutDisseminatio.pdf).
	Effective dissemination strategies	CDC collaborated with the National Association of Chronic Disease Directors to support a multi-site evaluation of state health department approaches to dissemination of evidence-based interventions. This evaluation documented the effectiveness of working with multi-site delivery systems, embedding interventions into routine operations, collaborating with other chronic disease programs and prioritizing the expansion of reach (Strategic approaches to expanding the reach of evidence-based interventions: results of a multi-state evaluation) (Unpublished report submitted to the National Association of Chronic Disease Directors by Westat, 2012.) (http://www.cdc.gov/arthritis/publications/reports.htm).

*^a^CDC funded these efforts unless otherwise noted*.

*^b^CDC provided partial funding*.

Through funded research and other mechanisms, CDC and its partners have employed a variety of strategies *to involve consumers* by using applied community-based participatory strategies in developing evidence-based programs and audience research. *To expand the range and reach of services*, CDC supports the development, evaluation, and dissemination of a variety of small group, tele-health, self-study, and online self-management support tools. *To advance evidence*, CDC investigators conduct systematic reviews of the literature and CDC funds applied prevention research to establish or strengthen the evidence-base of programs and policies. *To improve the effectiveness and appropriateness of services*, CDC supports comparative effectiveness studies and research designed to develop and test clinical-decision support tools. In terms of efforts *to strengthen inter-sector linkages*, CDC supports community-clinical collaborations and makes linkages across public health sectors. CDC also helps *to foster multi-sector commitment and accountability* through the development of new frameworks and guidelines. Finally, CDC invests *in building infrastructure* to deliver self-management support intervention programs at the national, state, and local levels systems initiatives.

## Sustaining Self-Management Support: Gaps and Opportunities

The need to advance efforts in self-management support is well recognized in the public health arena. However, challenges remain and several research questions are yet unanswered. Such questions include how to identify the essential elements of an intervention, how to best target effective interventions to specific audiences, and how to determine the effect of self-management support on critical public health outcomes and biometric measures such as hemoglobin A1c and blood pressure. Additional comparative effectiveness and cost effectiveness research studies of self-management support interventions are necessary. Importantly, selected papers in this special issue will help address these issues.

If self-management support interventions are to achieve their potential for public health impact, they need to be integrated into comprehensive chronic disease management strategies at the national, state, and local levels, and across sectors. Given the large and diverse population of people living with chronic conditions, engagement of multiple organizations across various sectors is required to reach those in need. Ideally, self-management support will become an integral element of clinical care standards of care (30), part of the routine menu of services offered by a variety of community agencies, and an essential component of community chronic disease control efforts. Finally, sustaining self-management support will require the infrastructure as well as multi-sectoral resources to reach people where they live, learn, work, and engage with their family and community. Creative financing mechanisms will need to be developed or expanded to ensure wide availability of evidence-based self-management support.

The Centers for Disease Control and Prevention is supporting a wide variety of self-management support activities across multiple strategic directions. CDC supported activities exemplify a comprehensive view of self-management support that encompasses both health-enhancing individual behaviors and physical and social environmental contexts that influence self-management behaviors. To advance self-management support, several important areas of research need to be conducted, broad-based organizational engagement needs to occur, and delivery capacity infrastructure and financing mechanisms need to be established or expanded. Despite these challenges, it remains essential to create self-management support services and environmental supports that allow people to live well with their chronic condition.

## Conflict of Interest Statement

The authors declare that the research was conducted in the absence of any commercial or financial relationships that could be construed as a potential conflict of interest.

This paper is included in the Research Topic, “Evidence-Based Programming for Older Adults.” This Research Topic received partial funding from multiple government and private organizations/agencies; however, the views, findings, and conclusions in these articles are those of the authors and do not necessarily represent the official position of these organizations/agencies. All papers published in the Research Topic received peer review from members of the Frontiers in Public Health (Public Health Education and Promotion section) panel of Review Editors. Because this Research Topic represents work closely associated with a nationwide evidence-based movement in the US, many of the authors and/or Review Editors may have worked together previously in some fashion. Review Editors were purposively selected based on their expertise with evaluation and/or evidence-based programming for older adults. Review Editors were independent of named authors on any given article published in this volume.
